# Habituation in *Frankliniella occidentalis* to deterrent plant compounds and their blends

**DOI:** 10.1111/eea.12187

**Published:** 2014-04-22

**Authors:** Barbara Egger, Bernhard Spangl, Elisabeth Helene Koschier

**Affiliations:** ^1^ Division of Crop ProtectionDepartment of Crop SciencesUniversity of Natural Resources and Life Sciences (BOKU)Peter‐Jordan‐Straße 821190ViennaAustria; ^2^ Institute of Applied Statistics and Computing (IASC)Department of Landscape, Spatial and Infrastructure SciencesUniversity of Natural Resources and Life Sciences (BOKU)Peter‐Jordan‐Straße 821190ViennaAustria

**Keywords:** Western flower thrips, secondary plant compound, feeding deterrence, methyl jasmonate, *cis*‐jasmone, allylanisole, Thysanoptera, Thripidae

## Abstract

Feeding and oviposition deterrence of three secondary plant compounds and their 1:1 blends to adult female *Frankliniella occidentalis *Pergande (Thysanoptera: Thripidae) and the potential for habituation of the thrips to the pure compounds and the 1:1 blends at various concentrations were investigated. In choice assays, we tested dose‐dependent feeding and oviposition deterrence of the two fatty acid derivatives methyl jasmonate and *cis*‐jasmone, the phenylpropanoid allylanisole, and their blends when directly applied to bean leaf discs. The concentration required to reduce the feeding damage by 50% relative to the control treatment (FDC_50_) was lowest for *cis*‐jasmone and highest for allylanisole. The feeding deterrent effect of both jasmonates was increased when blended with allylanisole. Feeding deterrence and oviposition deterrence were strongly correlated. In no‐choice assays conducted over four consecutive days, we discovered that dilutions at low concentrations (FDC_15_) applied to bean leaves resulted in habituation to the deterrents, whereas no habituation occurred at higher concentrations (FDC_50_). We observed a tendency that the 1:1 blends reduce the probability that thrips habituate to the deterrent compounds. Our results may be useful in the development of integrated crop protection strategies with the implementation of allelochemicals as pest behaviour‐modifying agents.

## Introduction

Western flower thrips, *Frankliniella occidentalis* Pergande (Thysanoptera: Thripidae), is one of the most harmful pests on many horticultural and agricultural crops worldwide (Kirk & Terry, 2003). Adults and immature stages both feed on plant tissue by penetrating plant cells and sucking out the cell sap (Childers, 1997). In addition to the direct feeding damage, plants are damaged indirectly because *F. occidentalis* is a potent vector of plant virus diseases (Wijkamp et al., 1995). Management of *F. occidentalis* is problematic due to their minute size and their thigmotactic behaviour (Lewis, 1997). Control strategies relying on repeated application of chemical insecticides have resulted in widespread development of resistance in *F. occidentalis* (e.g., Jensen, 2000). Among possible approaches to thrips control, the integration of secondary plant compounds that disrupt host acceptance behaviours with other control measures into behavioural manipulation strategies is broadly considered to have great potential (Cowles, 2004; Cook et al., 2007).

Plants produce a wide range of secondary compounds that may act as allelochemicals, mediating interactions between insects and plants. Some volatile secondary metabolites act as insect behaviour‐modifying agents or are toxic to various insect species (Renwick, 1999; Kim & Ahn, 2001). Allylanisole or estragole, a volatile phenylpropanoid (Knudsen et al., 1993), is a compound found in the essential oil of *Pimpinella anisum* L. and *Ocimum basilicum* L. (Hasegawa et al., 1997; Santos et al., 1998). The essential oil of *Foeniculum vulgare* Mill. also contains allylanisole and was deterrent to some beetle species (Cosimi et al., 2009). *cis*‐Jasmone and methyl jasmonate, two volatile fatty acid derivatives (Knudsen et al., 1993), are constituents of various essential oils, for instance from *Jasminum*,* Lonicera*, and *Philadelphus* species (Joulain, 1986; Mookherjee et al., 1990). Being stress‐related secondary plant compounds, both jasmonates are known to play a role in plant defence against herbivores as well (Birkett et al., 2000; Howe & Jander, 2008). Jasmonates were found to be repellent to various aphid species (Birkett et al., 2000; Bruce et al., 2003). Previous findings indicate that *F. occidentalis* respond negatively to jasmonates: females avoided settling and feeding on methyl jasmonate‐treated chrysanthemum plants (Bruhin, 2009), significantly fewer thrips were found on jasmonic acid‐sprayed plants (Thaler et al., 2001), and jasmonate‐baited traps did not attract *F. occidentalis* (James, 2005).

Habituation is the waning of a response as a result of repeated presentation of a stimulus (Chapman & Bernays, 1989; Schoonhoven et al., 2005). This type of experience‐based response has been found to occur in phytophagous insects for feeding deterrents and may reduce the effect of behavioural pest control strategies relying on behavioural manipulation of the pest (Jermy et al., 1982; Jermy, 1987; Glendinning & Gonzalez, 1995; Akhtar & Isman, 2003, 2004). Generally, the potential for habituation may be greater in polyphagous species such as *F. occidentalis,* presumably because they have evolved mechanisms for dealing with plant defensive compounds (Bernays & Chapman, 1994; Bernays et al., 2000). Whether – and to what extent – habituation to plant compounds can develop may also depend on the compound concentration and mixture. Habituation to feeding deterrents applied to plants occurs most readily when a single pure compound provides a weak inhibitory stimulus (Szentesi & Bernays, 1984; Held et al., 2001), whereas complex mixtures of antifeedants can prevent a decrease in feeding deterrent responses (Jermy, 1987; Bomford & Isman, 1996; Renwick & Huang, 1996). Strategies such as the mixture of several deterrents have been shown to have potential for mitigating the decrease in feeding deterrent responses to antifeedants by insects (Akhtar & Isman, 2003).

Here, we study habituation effects of deterrents in a cell sap‐feeding insect species with piercing‐sucking mouthparts. This study investigates the little‐researched responses of adult insects to feeding and oviposition deterrents (Held et al., 2001; Akhtar & Isman, 2004; Liu et al., 2005; Wang et al., 2008). Specifically, we investigate possible deterrent effects of three pure essential oil compounds and their binary (1:1) mixtures, applied directly to bean leaf discs, on adult female *F. occidentalis* and the potential for habituation of the thrips to the pure and mixed compounds.

## Materials and methods

### Insects and plants

A greenhouse‐collected strain of *F. occidentalis* was reared on detached bean leaves [*Phaseolus vulgaris* L. cv. Borlotto (Fabaceae); Austrosaat, Vienna, Austria] on 1% (wt/vol) water agar (Agar; Sigma‐Aldrich, Vienna, Austria) in plastic Petri dishes (14 cm diameter) in a climate chamber at 24 ± 1 °C, 35 ± 5% r.h., and L16:D8 photoperiod. About 50 adult females were allowed to lay eggs on bean leaves in the Petri dishes. The dishes were closed with lids with central holes covered with a fine mesh to ensure ventilation. After 48 h, the thrips were removed and the leaves with eggs were kept in Petri dishes in the climate chamber until adults emerged. Adults were used to maintain rearing or for the bioassays.

To obtain groups of even‐aged thrips females, thrips pupae were collected from the rearing and transferred to fresh bean leaves on 1% water agar in separate Petri dishes. Females were used in all bioassays 48 h after adult emergence, i.e., at the end of their pre‐oviposition period (van Rijn et al., 1995).

Bean plants used for rearing as well as for testing were grown in a plant growing room at 25 ± 1 °C, at 50 ± 5% r.h., and L16:D8 photoperiod in groups of 13–15 plants per pot. Leaf discs used for the bioassays were punched with a cork borer (1.1 cm diameter) from cotyledons of bean plants 11–13 days after sowing.

### Pure compounds and their blends

The pure compounds *cis*‐jasmone and methyl jasmonate were purchased from Sigma‐Aldrich, and allylanisole from Merck (Darmstadt, Germany). All pure compounds were blended at a ratio of 1:1 (wt/wt), i.e., each compound represents half of the total dose tested. Pure compounds as well as their three blends were diluted in pure ethanol (Merck) at a ratio of 1:10 by volume. Subsequently, the respective amount of distilled water plus Triton X‐100 (0.05%; Sigma‐Aldrich) as surfactant was added to obtain a range of concentrations (0.1–2%). The control solution consisted of ethanol and distilled water with the surfactant at a ratio of 1:10.

### General bioassay procedure

Bean leaf discs were put in a glass Petri dish (9 cm diameter) and sprayed with the respective dilution using a Potter Precision Laboratory Spray Tower (Burkard Manufacturing, Rickmansworth, UK) at constant air pressure resulting in an exact wet deposit of 1 μl cm^−2^ dilution quantity on the upper surface of each leaf disc. Before releasing the test insects, the treated leaf discs were allowed to dry for ca. 10 min and subsequently placed on a 1% water agar layer in glass Petri dishes (6 cm diameter) to prevent the thrips from feeding on the lower leaf surfaces. The units were closed with a plastic sealing film (Carl Roth, Karlsruhe, Germany) which was perforated subsequently by means of insect pins (ca. 10 punctures cm^−2^) to ensure ventilation.

### Feeding and oviposition deterrence index

In a choice assay adapted from Bomford & Isman (1996), a bean leaf disc treated with the control dilution and a leaf disc treated with a compound or blend dilution were placed at a distance of ca. 4 cm to each other on a thin 1% water agar layer in a glass Petri dish (6 cm diameter) to prevent the thrips from feeding on the lower leaf surfaces. A single *F. occidentalis* female of known age was released in the centre of the Petri dish. The dish was sealed with perforated plastic sealing film as described above and kept in a climate chamber at 24 ± 1 °C, 35 ± 5% r.h., and L16:D8 photoperiod. After 24 h, the female was removed, the area of feeding damage on each leaf disc was measured using a transparent counting grid (0.25 × 0.25 mm; Boraident, Halle, Germany) and a stereo microscope (Stemi 2000; Zeiss, Vienna, Austria). Subsequently, the eggs on each leaf disc were counted using a transmitted light microscope. This procedure was repeated with 4–6 concentrations (0.1, 0.25, 0.5, 0.75, 1, and 2%) of each compound or blend dilution for calculating a feeding or oviposition deterrence index (FDI or ODI), using the formulae FDI = 100 [(FC − FT)/(FC + FT)], where FC and FT are the control and treated leaf areas damaged by the *F. occidentalis* females (Isman et al., 1990), and ODI = 100 [(OC − OT)/(OC + OT)], where OC and OT are the numbers of eggs laid on the control and treated leaf discs, respectively.

The bioassay was replicated with 25–35 thrips females per concentration of each compound or blend dilution. FDC_15_, FDC_50_, or FDC_95_ (that is, the concentration required to reduce feeding damage on the treated leaf disc by 15, 50, or 95% relative to the control leaf disc) was calculated via linear regression (Seffrin et al., 2010).

### Repeated short‐term exposure

In a no‐choice assay adapted from Bomford & Isman (1996), *F. occidentalis* females were tested for habituation to the pure compounds and their 1:1 blends compared to the control dilution applied to bean leaf discs. The pure compounds were tested at their FDC_15_ and FDC_50_, and *cis*‐jasmone was tested additionally at the FDC_95_ (see Table 1 for concentrations). The blends were tested at their FDC_50_.

**Table 1:** Concentrations (%) of pure compounds and their 1:1 blends required to produce 50% feeding (FDC_50_) or oviposition (ODC_50_) deterrence in *Frankliniella occidentalis* on bean. Calculations by means of linear regression are based on 4–5 concentrations (n = 25–35 thrips females each) per compound/blend

Pure compound/blend	Feeding deterrence		Oviposition deterrence	
	FDC_50_	r^2^	ODC_50_	r^2^
*cis‐*Jasmone	0.66	0.86	0.67	0.83
Methyl jasmonate	0.77	0.99	0.60	0.97
Allylanisole	0.94	0.97	0.96	0.99
Methyl jasmonate/*cis*‐jasmone	0.66	0.68	0.71	0.63
Methyl jasmonate/allylanisole	0.31	0.88	0.29	0.96
*cis‐*Jasmone/allylanisole	0.51	0.89	0.58	0.98

At 15:00 hours of the 1st day of the bioassay, a *F. occidentalis* female of known age was placed singly on an untreated leaf disc on water agar in a glass Petri dish as described above and stored in a climate chamber at 24 ± 1 °C, 35 ± 5% r.h., and L16:D8 photoperiod. At 09:00 hours on the following day, the adult female was transferred to a leaf disc treated either with the compound or blend dilution or the control dilution in a glass Petri dish as described above and put back into the climate chamber. Six hours later the female was again transferred to a fresh untreated leaf disc and allowed to feed for 18 h. This procedure was repeated so that each female was exposed to the same treatment for 6 h on each of four consecutive days. Immediately after each transfer of the thrips, the feeding damage was measured and the eggs were counted on the leaf discs as described above. The bioassay was replicated with 23–34 *F. occidentalis* females for each compound or blend dilution at the respective FDC.

### Statistical analysis

The correlation between feeding deterrence and oviposition deterrence was determined by calculating Pearson's correlation coefficient for the data obtained in the feeding/oviposition deterrence choice assay. Data obtained in the assays with repeated short‐term exposures of thrips to pure compounds or their blends, i.e., the mean area damaged by thrips feeding and the mean number of eggs laid on the leaf discs, were analysed using repeated measures ANOVA, separating the means on the consecutive treatment days by Bonferroni post‐hoc tests. Degrees of freedom were corrected using Greenhouse‐Geisser estimates if the assumption of sphericity was violated (Mauchly's test for sphericity). Mean feeding damage/oviposition rates of the test compound treatments were compared to the control treatment using a one‐way ANOVA combined with a Bonferroni or a Games‐Howell post‐hoc test for pairwise comparisons of means. All statistical analyses were performed using the statistical package PASW 18.0.0 (IBM‐SPSS, Armonk, NY, USA).

## Results

### Feeding and oviposition deterrence

**Figure 1:**
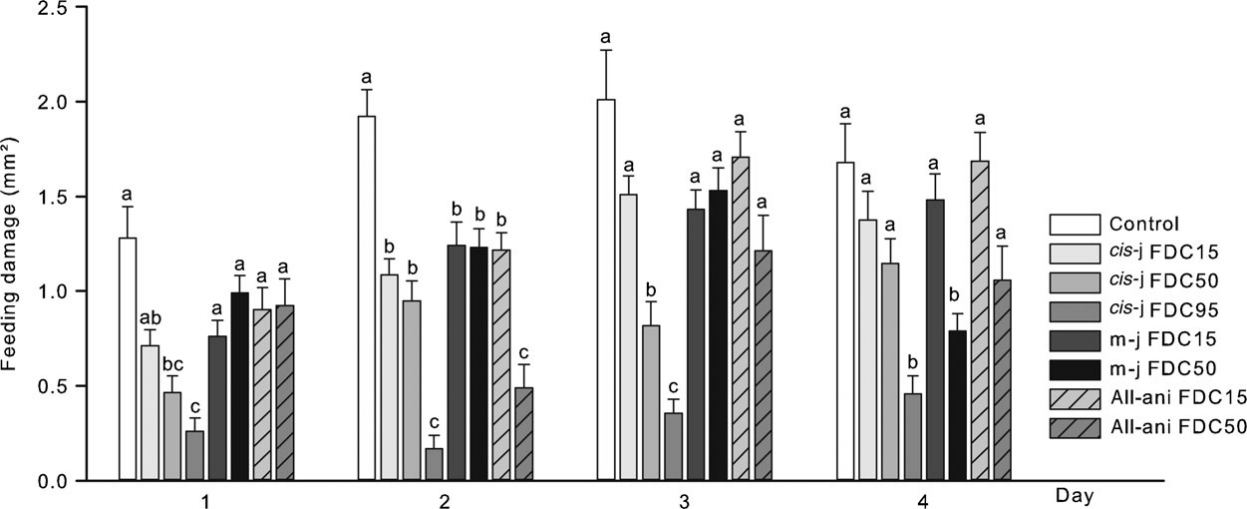
Mean (A) feeding deterrence and (B) oviposition deterrence of increasing concentrations of the pure compounds *cis*‐jasmone (*cis*‐j), methyl jasmonate (m‐j), and allylanisole (all‐ani), and the 1:1 blends *cis*‐jasmone/methyl jasmonate, *cis*‐jasmone/allylanisole, and methyl jasmonate/allylanisole to *Frankliniella occidentalis* females on bean leaf discs in choice assays (n = 25–30 thrips females per compound and concentration tested).

Generally, in all bioassays the feeding deterrence of pure compounds as well as their 1:1 blends was significantly correlated with the respective oviposition deterrence to *F. occidentalis* on bean leaves (Pearson correlation: r = 0.93, d.f. = 812, P<0.01; Figure 1). The feeding and oviposition deterrent effect on *F. occidentalis* of the pure compounds methyl jasmonate, *cis*‐jasmone, and allylanisole as well as their 1:1 blends methyl jasmonate/*cis*‐jasmone, methyl jasmonate/allylanisole, and *cis*‐jasmone/allylanisole (Figure 1) applied to leaf discs was dose‐dependent: increasing concentrations resulted in reduced feeding damage and reduced oviposition rate. The application of *cis*‐jasmone at about 0.66% reduced the feeding damage by 50% on the treated leaf disc compared to the control disc, methyl jasmonate applied at about 0.77% was required to deter thrips feeding by 50%, and allylanisole at about 0.94% reduced feeding damage by 50% (Table 1). The 1:1 blends of allylanisole and methyl jasmonate or *cis*‐jasmone deterred thrips from feeding at lower concentrations than the jasmonates applied as pure compounds: FDC_50_ of the 1:1 blend methyl jasmonate/allylanisole was about 0.31%, FDC_50_ of *cis*‐jasmone/allylanisole was about 0.51%. The 1:1 blend methyl jasmonate/*cis*‐jasmone deterred feeding by 50% when applied at about 0.66%. The concentration required to deter oviposition by 50% was about 0.67% for *cis*‐jasmone, 0.60% for methyl jasmonate, and 0.96% for allylanisole. The 1:1 blend of methyl jasmonate/*cis*‐jasmone reduced oviposition by 50% at about 0.71%, methyl jasmonate/allylanisole at about 0.29%, and *cis*‐jasmone/allylanisole at about 0.58%.

### Repeated short‐term exposure

#### Effects within exposure days

**Figure 2:**
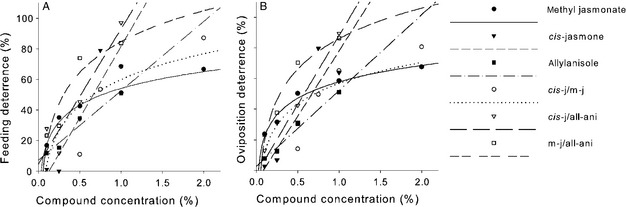
Mean (+ SEM) feeding damage caused by *Frankliniella occidentalis* during a 6‐h exposure period on four consecutive days on treated bean leaf discs in a no‐choice assay. Bean leaves were treated with ethanol + water (control), *cis*‐jasmone (*cis*‐j) at 0.29% (FDC_15_), 0.66% (FDC_50_), or 1.15% (FDC_95_), methyl jasmonate (m‐j) at 0.08% (FDC_15_) or 0.77% (FDC_50_), or allylanisole (all‐ani) at 0.17% (FDC_15_) or 0.94% (FDC_50_) (n = 24–34 thrips females per dilution tested). Means within a day capped with different letters are significantly different (one‐way ANOVA, followed by Bonferroni pairwise comparisons: P<0.05).

The application of *cis*‐jasmone at the FDC_15_ (0.29%) reduced feeding damage caused by *F. occidentalis* females during the 6‐h exposure period on day 2, but on days 1, 3, and 4 no significant reduction in the feeding damage compared to the control treatment was found (Figure 2). *cis‐*Jasmone at the FDC_50_ (0.66%) deterred *F. occidentalis* from feeding on days 1–3. On day 4, no significant reduction in the feeding damage on treated leaf discs was found. *cis‐*Jasmone at the FDC_95_ (1.15%) deterred the thrips within all exposure days compared to the control treatment.

Methyl jasmonate or allylanisole applied at the respective FDC_15_ (0.08 or 0.17%) to bean leaf discs reduced feeding damage during the 6‐h exposure period on day 2, but not on days 1, 3, and 4 compared to the control treatment. Methyl jasmonate at the FDC_50_ (0.77%) deterred *F. occidentalis* females on days 2 and 4. No significant reduction in the feeding damage on treated leaf discs compared to the control discs was found on days 1 and 3. Allylanisole at the FDC_50_ (0.94%) deterred *F. occidentalis* females on day 2, on the other days, no significant reduction in the feeding damage on treated leaf discs compared to the control discs was found during 6 h of exposure.

**Figure 3:**
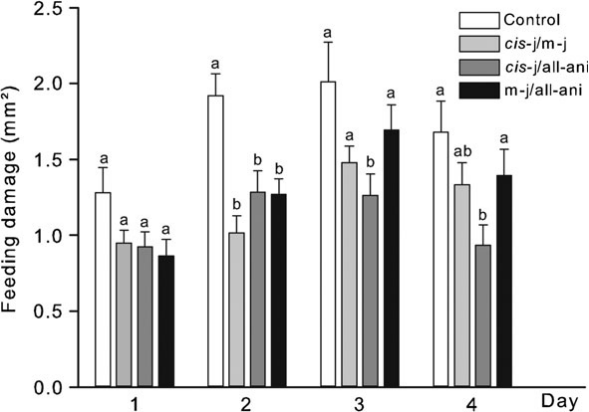
Mean (+ SEM) feeding damage caused by *Frankliniella occidentalis* during a 6‐h exposure period on four consecutive days on treated bean leaf discs in a no‐choice assay. Bean leaves were treated either with ethanol + water (control), the binary mixtures methyl jasmonate/*cis*‐jasmone (m‐j/*cis*‐j) at 0.66% (FDC_50_), *cis*‐jasmone/allylanisole (*cis*‐j/all‐ani) at 0.51% (FDC_50_), or methyl jasmonate/allylanisole (m‐j/all‐ani) at 0.31% (FDC_50_) (n = 24–34 thrips females per dilution tested). Means within a day capped with different letters are significantly different (one‐way ANOVA, followed by Bonferroni pairwise comparisons: P<0.05).

Applications of the 1:1 blends of methyl jasmonate/*cis*‐jasmone or methyl jasmonate/allylanisole at the respective FDC_50_ (0.66 or 0.31%) reduced the feeding damage of *F. occidentalis* females during the 6‐h exposure period significantly compared to the control treatment on day 2, but on days 1, 3, and 4 no significant reduction in the feeding damage on treated leaf discs was found (Figure 3). The 1:1 blend *cis*‐jasmone/allylanisole applied at FDC_50_ (0.51%) reduced the feeding damage significantly on days 2, 3, and 4, but not on day 1 in within‐day comparisons to the control treatment.

#### Effects between exposure days

Generally, the feeding activity of *F. occidentalis* resulting in damaged areas on control bean leaf discs varied significantly during the 6‐h exposure period among the four consecutive days (ANOVA for repeated measures: F_3,72_ = 3.64, P = 0.017; Figure 2). Bonferroni post‐hoc pairwise comparisons between days revealed that thrips feeding on the control discs significantly increased on day 2 compared to day 1, but on days 3 and 4 feeding did not differ statistically from days 1 and 2.

Between‐day comparisons revealed that the feeding activity of thrips on leaf discs treated with *cis*‐jasmone at the FDC_15_ increased significantly from day 1 to day 2, and on day 3 compared to days 1 and 2. On day 4 feeding differed statistically from day 1, but not from days 2 and 3 (F_3,75_ = 13.74, P = 0.001). On leaf discs treated with *cis*‐jasmone at the FDC_50_, feeding damage increased significantly from day 1 to day 2, but there was no difference between areas of feeding damage on days 2–4 (F_3,84_ = 10.71, P<0.001). Feeding damage on discs treated with *cis*‐jasmone at the FDC_95_ did not significantly increase on four consecutive days (F_3,69_ = 2.80, P = 0.051).

The deterrent effect of methyl jasmonate in between‐day comparisons at the lower concentration (FDC_15_) gradually decreased: the amount of feeding damage was significantly lower on day 1 compared to the following 3 days (F_3,75_ = 15.01, P>0.001). On leaf discs treated with methyl jasmonate at the FDC_50_, feeding damage increased significantly from day 1 to day 3, but on day 4 feeding damage decreased significantly compared to days 2 and 3 (F_3,72_ = 13.66, P<0.001).

Between‐day comparisons showed that the feeding activity of thrips on leaf discs treated with allylanisole at the FDC_15_ increased significantly going from day 1 to day 3. The feeding damage on day 4 was not significantly different from that on day 3 (F_3,66_ = 13.82, P<0.001). On leaf discs treated with allylanisole at FDC_50_, feeding damage did not increase. On day 3, the damage increased significantly compared to day 2, the damaged area on day 4 did not differ from that on days 1–3 (F_2.01,50.34_ = 3.90, P = 0.026).

Between‐day comparisons of methyl jasmonate/*cis*‐jasmone‐treated leaf discs revealed a significantly increased feeding activity on days 3 and 4 (F_3,66_ = 7.56, P<0.001; Figure 3). Comparing the feeding activity of thrips on leaf discs treated with methyl jasmonate/allylanisole at the FDC_50_ on the four consecutive exposure days revealed that the feeding damage increased significantly on day 2 compared to day 1, and on day 3 compared to days 1 and 2. On day 4, the feeding damage did not increase compared to day 3 (F_3,75_ = 10.64, P<0.001). Bonferroni post‐hoc tests resulted in no significant differences in the feeding damage on leaf discs treated with the 1:1 blend *cis*‐jasmone/allylanisole at FDC_50_ (0.51%) over 4 days in between‐day comparisons, although the ANOVA for repeated measures indicated a significant treatment effect (F_3,66_ = 2.96, P = 0.039).

## Discussion

Based on the FDC_50_ values, *cis*‐jasmone deterred thrips feeding by 50% at the lowest concentration, followed by methyl jasmonate and allylanisole. The latter was chosen for the bioassays because it belongs to a different chemical class than the jasmonates tested and because of the repellent properties of fennel essential oil and allylanisole against various beetle species (Knudsen et al., 1993; Hayes et al., 1994; Cosimi et al., 2009). It was identified as a thrips deterrent compound for the first time.

As a pure compound, allylanisole displayed a relatively high FDC_50_ (0.94%) compared to the FDC_50_ of the pure jasmonates, whereas in blends the FDC_50_ were comparatively low: methyl jasmonate/allylanisole (0.31%) and *cis*‐jasmone/allylanisole (0.51%). The blend of the two jasmonates did not show a noticeable increase in deterrent effect compared to the pure compounds. Blending of a jasmonate—belonging to the class of fatty acid derivatives—and allylanisole—a phenylpropanoid (Knudsen et al., 1993)—apparently increased deterrence efficiency. Binary and complex mixtures were reported to be more effective compared to single compounds as deterrents to other insect species, e.g., *Locusta migratoria* L., *Sitophilus granarius* L., or *Spodoptera litura* Fabricius (Adams & Bernays, 1978; Castellanos & Espinosa‐García, 1997; Hummelbrunner & Isman, 2001).

The no‐choice assay for habituation on four consecutive days showed an increased feeding damage on days 2 and 3 in the control treatment, which dropped on day 4. A possible explanation could be the natural reproductive cycle of *F. occidentalis* females: the oviposition rate peaks 3 days after adult emergence, thus causing the necessity of enhanced food uptake (van Rijn et al., 1995). Feeding and oviposition deterrence were strongly correlated in all bioassays, independently from the treatment. This matches the decreasing reproductive fitness as a result of minor food quality as shown in a study with *F. occidentalis* by Trichilo & Leigh (1988).

We assessed the potential of *F. occidentali*s to habituate to secondary plant compounds in repeated exposure bioassays using the deterrent *cis*‐jasmone at various concentrations as a model compound. Weak stimuli induced habituation, whereas strong stimuli did not, supporting the results of previous studies of Jermy et al. (1982) and Szentesi & Bernays (1984): the lowest concentration of *cis*‐jasmone (FDC_15_ = 0.29%) was deterrent only during the first half of the 4‐day testing period, the intermediate concentration (FDC_50_ = 0.66%) deterred the thrips over a longer period and the highest concentration (FDC_95_ = 1.15%) did not decrease the deterrent efficiency over all 4 days tested. Methyl jasmonate and allylanisole applied as pure compounds at low (FDC_15_) and intermediate (FDC_50_) concentrations supported the thesis that habituation is dose‐dependent in thrips.

We observed a tendency that thrips needed a longer exposure to habituate to the 1:1 blends at the respective FDC_50_ of the compounds. However, this was not always the case: the two jasmonates applied as single compounds at the respective FDC_50_ deterred thrips feeding more effectively compared to allylanisole, whereas the 1:1 blend of the jasmonates was deterrent to the thrips only on day 2. The same applies to the blend methyl jasmonate/allylanisole; the blend *cis*‐jasmone/allylanisole exhibited a deterrent effect on 3 of 4 days. Considering that the quantity of either jasmonate is reduced in their binary mixture, we suppose that this reduced quantity of either deterrent enhances the probability for habituation in thrips. Habituation might occur in bioassays with the two jasmonates in mixture because the two compounds are chemically closely related. In contrast, the blend *cis*‐jasmone/allylanisole inhibited habituation, although the blend concentration was even lower (0.51%). The prolonged deterrent effect of this blend may be due to the increased complexity of the blend and the presence of *cis*‐jasmone, the most effective pure deterrent compound tested. However, further research using more and other mixtures is required to better understand the mode of action of the compounds. Methyl jasmonate, however, may be too weak a deterrent stimulus at the low concentration in the binary mixture with allylanisole to maintain the deterrent effect over the 4‐day testing period. Our results correspond to previous studies about the correlation between mixture complexity and habituation potential in insects (Jermy, 1987; Bomford & Isman, 1996).

This study is the first to investigate the habituation potential of a generalist, cell sap‐feeding insect with piercing‐sucking mouthparts to secondary plant compounds. The compounds tested remained deterrent over several days when applied at their respective FDC_50_. Application at lower concentrations (FDC_15_) resulted in habituation of the thrips to the pure compounds. The amount of a pure compound used to deter *F. occidentalis* may be reduced by blending deterrent agents, thus enhancing the chemical complexity and preventing habituation to the compounds. It should be noted that a direct application of jasmonates to crop plants also might induce defence mechanisms in the plant (Farmer & Ryan, 1990; Browse & Howe, 2008). These effects might be considered in further studies. Further experiments should evaluate the long‐term habituation potential of thrips before our results may be used in the development of integrated crop protection strategies with the implementation of allelochemicals as pest behaviour‐modifying agents.

## References

[eea12187-bib-0001] Adams CM & Bernays EA (1978) Effect of combinations of deterrents on feeding‐behavior of *Locusta migratoria*. Entomologia Experimentalis et Applicata 23: 101–109

[eea12187-bib-0002] Akhtar Y & Isman MB (2003) Binary mixtures of feeding deterrents mitigate the decrease in feeding deterrent response to antifeedants following prolonged exposure in the cabbage looper, *Trichoplusia ni* (Lepidoptera: Noctuidae). Chemoecology 13: 177–182

[eea12187-bib-0003] Akhtar Y & Isman MB (2004) Feeding responses of specialist herbivores to plant extracts and pure allelochemicals: effects of prolonged exposure. Entomologia Experimentalis et Applicata 111: 201–208

[eea12187-bib-0004] Bernays EA & Chapman RF (1994) Host‐Plant Selection by Phytophagous Insects. Chapman & Hall, New York, NY, USA

[eea12187-bib-0005] Bernays EA, Oppenheim S, Chapman RF, Kwon H & Gould F (2000) Taste sensitivity of insect herbivores to deterrents is greater in specialists than in generalists: a behavioral test of the hypothesis with two closely related caterpillars. Journal of Chemical Ecology 26: 547–563

[eea12187-bib-0006] Birkett MA, Campbell CAM, Chamberlain K, Guerrieri E, Hick AJ et al. (2000) New roles for *cis*‐jasmone as an insect semiochemical and in plant defense. Proceedings of the National Academy of Sciences of the USA 97: 9329–9334 10900270 10.1073/pnas.160241697 PMC16867

[eea12187-bib-0007] Bomford MK & Isman MB (1996) Desensitization of fifth instar *Spodoptera litura* to azadirachtin and neem. Entomologia Experimentalis et Applicata 81: 307–313

[eea12187-bib-0008] Browse J & Howe GA (2008) New weapons and a rapid response against insect attack. Plant Physiology 146: 832–838 18316637 10.1104/pp.107.115683 PMC2259070

[eea12187-bib-0009] Bruce TJA, Martin JL, Pickett JA, Pye BJ, Smart LE & Wadhams LJ (2003) *cis*‐Jasmone treatment induces resistance in wheat plants against the grain aphid, *Sitobion avenae* (Fabricius) (Homoptera: Aphididae). Pest Management Science 59: 1031–1036 12974355 10.1002/ps.730

[eea12187-bib-0010] Bruhin D (2009) Direct and Indirect Effects of Methyl Salicylate and Methyl Jasmonate on Frankliniella occidentalis Pergande on Pot Chrysanthemum. MSc Thesis, Universität für Bodenkultur, Vienna, Austria

[eea12187-bib-0011] Castellanos I & Espinosa‐García FJ (1997) Plant secondary metabolite diversity as a resistance trait against insects: a test with *Sitophilus granarius* (Coleoptera: Curculionidae) and seed secondary metabolites. Biochemical Systematics and Ecology 25: 591–602

[eea12187-bib-0012] Chapman RF & Bernays EA (1989) Insect behavior at the leaf surface and learning as aspects of host plant selection. Experientia 45: 215–222

[eea12187-bib-0013] Childers CC (1997) Feeding and oviposition injuries to plants Thrips as Crop Pests (ed. by T Lewis), pp. 505–537 CAB International, Wallingford, UK

[eea12187-bib-0014] Cook SM, Khan ZR & Pickett JA (2007) The use of push‐pull strategies in integrated pest management. Annual Review of Entomology 52: 375–400 10.1146/annurev.ento.52.110405.091407 16968206

[eea12187-bib-0015] Cosimi S, Rossi E, Cioni PL & Canale A (2009) Bioactivity and qualitative analysis of some essential oils from Mediterranean plants against stored‐product pests: evaluation of repellency against *Sitophilus zeamais* Motschulsky, *Cryptolestes ferrugineus* (Stephens) and *Tenebrio molitor* (L.). Journal of Stored Products Research 45: 125–132

[eea12187-bib-0016] Cowles RS (2004) Manipulation of host‐finding and acceptance behaviours in insects: importance to IPM Integrated Pest Management: Potential, Constraints and Challenges (ed. by O Koul, GS Dhaliwal & GW Cuperus), pp. 185–204 CABI Publishing, Wallingford, UK

[eea12187-bib-0017] Farmer EE & Ryan CA (1990) Interplant communication: airborne methyl jasmonate induces synthesis of proteinase inhibitors in plant leaves. Proceedings of the National Academy of Sciences of the USA 87: 7713–7716 11607107 10.1073/pnas.87.19.7713 PMC54818

[eea12187-bib-0018] Glendinning JI & Gonzalez NA (1995) Gustatory habituation to deterrent allelochemicals in a herbivore: concentration and compound specificity. Animal Behaviour 50: 915–927

[eea12187-bib-0019] Hasegawa Y, Tajima K, Toi N & Sugimara Y (1997) Characteristic components found in the essential oil of *Ocimum basilicum* L. Flavour and Fragrance Journal 12: 195–200

[eea12187-bib-0020] Hayes JL, Strom BL, Roton LM & Ingram LL (1994) Repellent properties of the host compound 4‐allylanisole to the southern pine‐beetle. Journal of Chemical Ecology 20: 1595–1615 24242654 10.1007/BF02059883

[eea12187-bib-0021] Held DW, Eaton T & Potter DA (2001) Potential for habituation to a neem‐based feeding deterrent in Japanese beetles, *Popillia japonica*. Entomologia Experimentalis et Applicata 101: 25–32

[eea12187-bib-0022] Howe GA & Jander G (2008) Plant immunity to insect herbivores. Annual Review of Plant Biology 59: 41–66 10.1146/annurev.arplant.59.032607.092825 18031220

[eea12187-bib-0023] Hummelbrunner LA & Isman MB (2001) Acute, sublethal, antifeedant, and synergistic effects of monoterpenoid essential oil compounds on the tobacco cutworm, *Spodoptera litura* (Lep., Noctuidae). Journal of Agricultural and Food Chemistry 49: 715–720 11262018 10.1021/jf000749t

[eea12187-bib-0500] Isman MB, Koul O, Luczynski A & Kaminski J (1990) Insecticidal and antifeedant bioactivities of neem oil and their relationship to azadirachtin content. Journal of Agricultural and Food Chemistry 38: 1406–1411

[eea12187-bib-0024] James DG (2005) Further field evaluation of synthetic herbivore‐induced plant volatiles as attractants for beneficial insects. Journal of Chemical Ecology 31: 481–495 15898496 10.1007/s10886-005-2020-y

[eea12187-bib-0025] Jensen SE (2000) Insecticide resistance in the western flower thrips, *Frankliniella occidentalis*. Integrated Pest Management Reviews 5: 131–146

[eea12187-bib-0026] Jermy T (1987) The role of experience in the host selection of phytophagous insects Perspectives in Chemoreception and Behavior (ed. by RF Chapman, EA Bernays & JGJ Stoffolano), pp. 143–157 Springer, New York, NY, USA

[eea12187-bib-0027] Jermy T, Bernays EA & Szentesi A (1982) The effect of repeated exposure to feeding deterrents on their acceptability to phytophagous insects 5th International Symposium on Insect‐Plant Relationships (ed. by JH Visser & AK Minks), pp. 25–32 Pudoc, Wageningen, The Netherlands

[eea12187-bib-0028] Joulain D (1986) Study of the fragrance given off by certain springtime flowers Progress in Essential Oil Research (ed. by E‐J Brunke), pp. 57–68 Walter de Gruyter, Berlin, Germany

[eea12187-bib-0029] Kim D‐H & Ahn Y‐J (2001) Contact and fumigant activities of constituents of *Foeniculum vulgare* fruit against three coleopteran stored‐product insects. Pest Management Science 57: 301–306 11455661 10.1002/ps.274

[eea12187-bib-0030] Kirk WDJ & Terry LI (2003) The spread of the western flower thrips *Frankliniella occidentalis* (Pergande). Agricultural and Forest Entomology 5: 301–310

[eea12187-bib-0031] Knudsen JT, Tollsten L & Bergstrom LG (1993) Floral scents – a checklist of volatile compounds isolated by headspace techniques. Phytochemistry 33: 253–280

[eea12187-bib-0032] Lewis T (1997) Thrips as Crop Pests. CAB International, Wallingford, UK

[eea12187-bib-0033] Liu SS, Li YH, Liu YQ & Zalucki MP (2005) Experience‐induced preference for oviposition repellents derived from a non‐host plant by a specialist herbivore. Ecology Letters 8: 722–729

[eea12187-bib-0034] Mookherjee BD, Trenkle RW & Wilson RA (1990) The chemistry of flowers, fruits and spices: live vs. dead – a new dimension in fragrance research. Pure and Applied Chemistry 62: 1357–1364

[eea12187-bib-0035] Renwick JAA (1999) Phytochemical modification of taste: an insect model Biologically Active Natural Products: Agrochemicals (ed. by HC Cutler & SJ Cutler), pp. 221–229 CRC Press, Boca Raton, FL, USA

[eea12187-bib-0036] Renwick JAA & Huang XP (1996) Development of sensitivity to feeding deterrents in larvae of *Pieris rapae*. Entomologia Experimentalis et Applicata 80: 90–92

[eea12187-bib-0037] van Rijn PCJ, Mollema C & Steenhuis‐Broers GM (1995) Comparative life history studies of *Frankliniella occidentalis* and *Thrips tabaci* (Thysanoptera: Thripiadae) on cucumber. Bulletin of Entomological Research 85: 285–297

[eea12187-bib-0038] Santos PM, Figueiredo AC, Oliveira MM, Barroso JG, Pedro LG et al. (1998) Essential oils from hairy root cultures and from fruits and roots of *Pimpinella anisum*. Phytochemistry 48: 455–460

[eea12187-bib-0039] Schoonhoven LM, van Loon JJA & Dicke M (2005) Insect‐Plant Biology, 2nd edn Oxford University Press, Oxford, UK

[eea12187-bib-0040] Seffrin RD, Shikano I, Akhtar Y & Isman MB (2010) Effects of crude seed extracts of *Annona atemoya* and *Annona squamosa* L. against the cabbage looper, *Trichoplusia ni* in the laboratory and greenhouse. Crop Protection 29: 20–24

[eea12187-bib-0041] Szentesi A & Bernays EA (1984) A study of behavioral habituation to a feeding deterrent in nymphs of *Schistocerca gregaria*. Physiological Entomology 9: 329–340

[eea12187-bib-0042] Thaler JS, Stout MJ, Karban R & Duffey SS (2001) Jasmonate‐mediated induced plant resistance affects a community of herbivores. Ecological Entomology 26: 312–324

[eea12187-bib-0043] Trichilo PJ & Leigh TF (1988) Influence of resource quality on the reproductive fitness of flower thrips (Thysanoptera, Thripidae). Annals of the Entomological Society of America 81: 64–70

[eea12187-bib-0044] Wang H, Guo W‐F, Zhang P‐J, Wu Z‐Y & Liu S‐S (2008) Experience‐induced habituation and preference towards non‐host plant odors in ovipositing females of a moth. Journal of Chemical Ecology 34: 330–338 18253797 10.1007/s10886-008-9433-3

[eea12187-bib-0045] Wijkamp I, Almarza N, Goldbach R & Peters D (1995) Distinct levels of specificity in thrips transmission of tospoviruses. Phytopathology 85: 1069–1074

